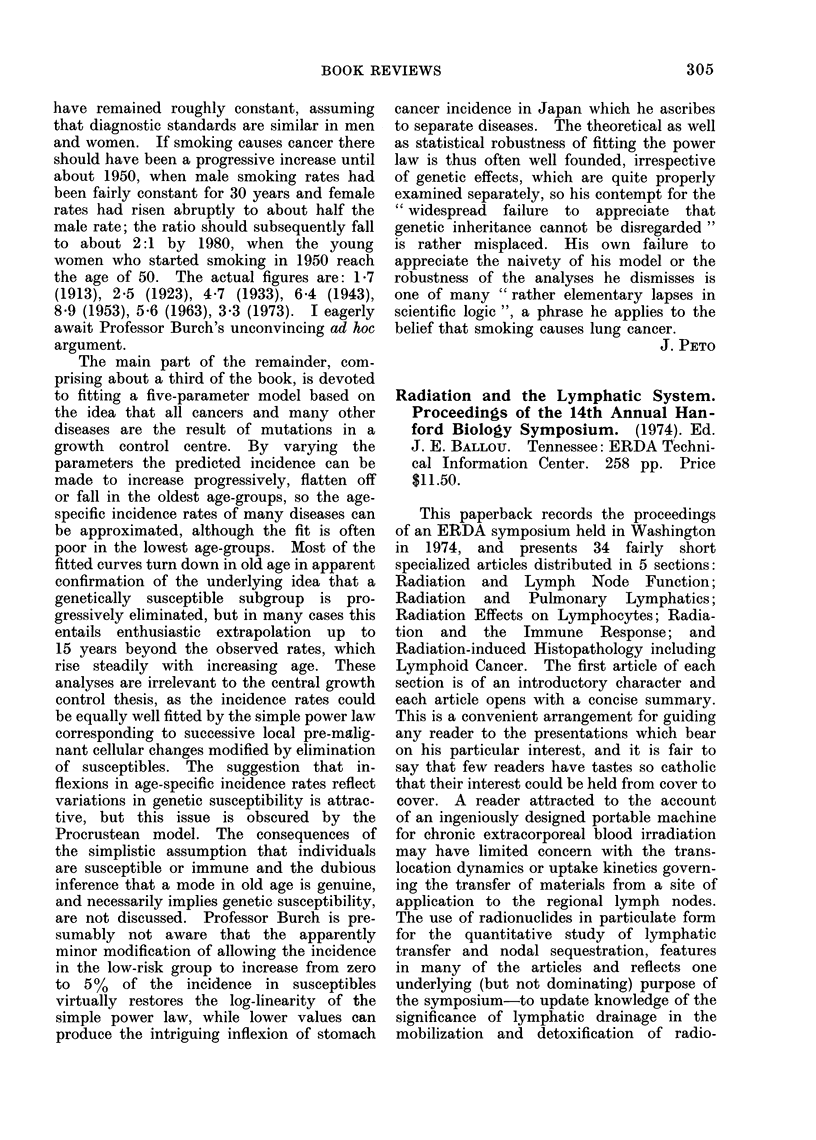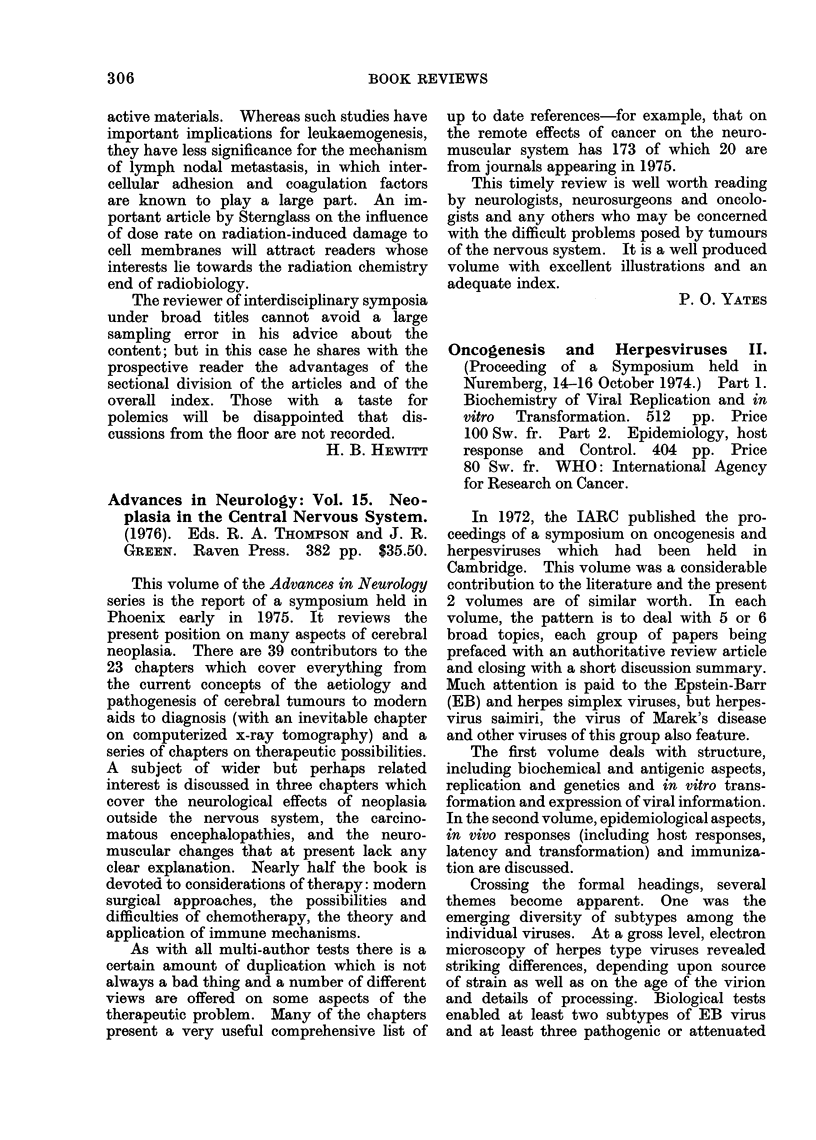# Radiation and the Lymphatic System. Proceedings of the 14th Annual Hanford Biology Symposium

**Published:** 1976-09

**Authors:** H. B. Hewitt


**Radiation and the Lymphatic System. Proceedings of the 14th Annual Hanford Biology Symposium.** (1974). Ed. J. E. Ballou. Tennessee: ERDA Technical Information Center. 258 pp. Price $11.50.

This paperback records the proceedings of an ERDA symposium held in Washington in 1974, and presents 34 fairly short specialized articles distributed in 5 sections: Radiation and Lymph Node Function; Radiation and Pulmonary Lymphatics; Radiation Effects on Lymphocytes; Radiation and the Immune Response; and Radiation-induced Histopathology including Lymphoid Cancer. The first article of each section is of an introductory character and each article opens with a concise summary. This is a convenient arrangement for guiding any reader to the presentations which bear on his particular interest, and it is fair to say that few readers have tastes so catholic that their interest could be held from cover to cover. A reader attracted to the account of an ingeniously designed portable machine for chronic extracorporeal blood irradiation may have limited concern with the translocation dynamics or uptake kinetics governing the transfer of materials from a site of application to the regional lymph nodes. The use of radionuclides in particulate form for the quantitative study of lymphatic transfer and nodal sequestration, features in many of the articles and reflects one underlying (but not dominating) purpose of the symposium—to update knowledge of the significance of lymphatic drainage in the mobilization and detoxification of radioactive materials. Whereas such studies have important implications for leukaemogenesis, they have less significance for the mechanism of lymph nodal metastasis, in which intercellular adhesion and coagulation factors are known to play a large part. An important article by Sternglass on the influence of dose rate on radiation-induced damage to cell membranes will attract readers whose interests lie towards the radiation chemistry end of radiobiology.

The reviewer of interdisciplinary symposia under broad titles cannot avoid a large sampling error in his advice about the content; but in this case he shares with the prospective reader the advantages of the sectional division of the articles and of the overall index. Those with a taste for polemics will be disappointed that discussions from the floor are not recorded.

H. B. Hewitt